# Supplementary material for: Professional competencies and public health content in the human nutrition and dietetics degree program: A qualitative study based on experts' consensus
Source: PLoS One. 2021 Jan 29;16(1):e0246268. doi: 10.1371/journal.pone.0246268 (PMC7845966; doi:10.1371/journal.pone.0246268)
Supplement: Attachment — Submitted filename: Comments.pdf [file pone.0246268.s001.pdf]

In this manuscript, the authors describe a consensus in the classification of professional competencies and basic public health content (already stated in Spain) to orientate the degree in Human Nutrition and Dietetic. In the consensus participated 14 lecturers from 11 Spanish universities which are part of the degree program of DHN. Results show competencies and basic content for training for the DHN. Author conclude that the *consensus* may be helpful as a reference to orient and update public health.

General: Overall, it is an interesting topic and very necessary to look into. The paper needs better clarity in several aspects. The introduction, author should give a clear and detailed background which will set the reader in the context, mainly because the nutritionist or dietitian profession is differently regulated in some countries and, and has different levels of development in the countries. In addition, aspects of the methodology need more detail, as how the consensus was reach, did the methodology included any instrument (validated)? The discussion, authors present their finding but the paper would be more interesting to read if there would be an analysis of what competencies actually imply in DHN programs.

Specific aspects:

1. The full proposed title is: “Professional Competencies and Public Health Content in the Diet and Human Nutrition Degree Program: a qualitative study based on experts' consensus”. However, in reference 19, 21 and 22 the degree program is “Diplomado en Nutrición Humana y Dietética”, it seems to me that DHN (referred by authors as Degree in Diet and Human Nutrition) should be Degree in Human Nutrition or Human Nutrition and Dietetic. This should be corrected in several parts of the article.

2. Some aspect are confusing or need to be clarified in the introduction. According to the article, Degree DHN forms Dietitians or Nutritionists, along the article, mainly in the discussion the term “dietary nutritionists” is mentioned. Is that a different term for referring a Dietitian or Nutritionist working in public health? Is a formal term?.

3. Because Dietitian/Nutritionist is regulated in different ways in different countries, I suggest to include on Introduction some local aspect of the profession like the existing areas in which Dietitian/Nutritionist are insert now (this is particular pertinent because later on the results some topics as Health risks associated with food products and Health audits and inspections are mentioned and those are mainly part of other areas related to manage of food system, staff and budget to provide safe and nutritious meals at hospitals/long term residences/collective feeding or Food services. Moreover, how it has been the profession´s development in the last years. It would be interesting to see a background of the DHN and its role in public health so far.

4. Regarding Methodology,
   1) References 7-10 gives a detailed of the steps, however more detail is needed in how the consensus was reached (Delphi panel, or other? Any validated instrument). Did authors checked/included any international experience or literature to know the role of Dietitian/Nutritionist in public health?.
   2) It would be important to clarify in details some characteristics of these group of 14 lecturers. Although is mentioned the categories of each of them (line 138) and also that professors had an experience in public health, is not clear if they were physicians, nurse, dietitian, etc. nor if any of them had a PhD in Nutrition Public Health?. I think is important to state if participants were highly qualified and with experience in public health related to food/ nutrition/disease/promotion/food safety/community nutrition/community education.
   3) Additionally, and very important, although competencies related to public health may be common to all public health practitioners; is necessary to describe discipline-specific

competencies for future dietitians/nutritionist professionals (discipline-specific competencies provide unique or technical skills, knowledge and abilities). Table 4 intent to do it but methodically is not clear how these competencies came up.

4. Regarding the 3 areas and list of 80 professional competencies in public health, defined for Spain. I have one comments. How different universities did define competencies in their respective programs? Was in this *consensus* any evaluation about how we are and we are we going?.
5. Regarding results section, it should be more analysis of generic and specific competencies, to emphasize what corresponds to health professionals and what to dietitian/nutritionist. A critical analysis of the literature or a state of the art of the current is required in order to identify generic and specific competencies in a DHN program. One of the most important objectives of the competencies is to discriminate relevant aspects from the general ones, to structure a curricular sequence of the contents in the programs.

6. Finally, It is important to discuss what is the importance of competencies like leadership, management, professionalism, communication and, ethics in the Nutritionist professionals. Are any of those considered? .

Other comments

- Line 124: when authors say “preferably from the area of preventive medicine and public health” which were the other areas that not were included?.
- Table 3, number 2: “Evaluation methods for population nutrition status”, should say “nutritional status”. Same for Analysis of data in nutrition studies (nutritional studies). Number 3: “Health problems and Dietary and Nutrition Strategies” (is should be nutritional strategies.
- The abstract mentions (line 57): “Fourteen lecturers in the degree program of DHN participated from 11 Spanish universities”; line 132 “14 ended up participating in the study (10 women and 4 men) from 11 universities” and line 198 “of 11 professors of the 15 public Spanish universities”.
- It is not clear the expression “ill-health prevention strategies..” (line 204).
