# Supplementary material for: Professional competencies and public health content in the human nutrition and dietetics degree program: A qualitative study based on experts' consensus
Source: PLoS One. 2021 Jan 29;16(1):e0246268. doi: 10.1371/journal.pone.0246268 (PMC7845966; doi:10.1371/journal.pone.0246268)
Supplement: Attachment — Submitted filename: Plos One comments_R1.pdf [file pone.0246268.s003.pdf]

All the comments have been addressed. However, let me suggest few more aspects.

1. Regarding the description of the group (Methods section, line 158). I understand that all the participants had a PhD. Also, in the group were doctors (n=5). Were those “doctors” Physicians? If that is correct, it should state (as Physician) to make clearer that were 5 Physicians and all the participants were PhD or hold doctorates.
2. Include the word diet in line 99: “of professionals prepared to use a public health approach to address problems related to diet and nutrition.
3. Line 217: number three should be replaced by the number (to follow the way how is written before)
4. Although discussion mention all the competencies that Dietitian/Nutritionist should addressed, authors also mention that many of those are multidisciplinary nature of the field. Few lines are necessary to state why this human (Dietitian/Nutritionist) resource is the most suitable to accomplish those activities (lines 300-305).
